# Case report: Extending the spectrum of clinical and molecular findings in FOXC1 haploinsufficiency syndrome

**DOI:** 10.3389/fgene.2023.1174046

**Published:** 2023-06-23

**Authors:** Alexandra Garza Flores, Ida Nordgren, Maria Pettersson, Dora Dias-Santagata, Daniel Nilsson, Anna Hammarsjö, Anna Lindstrand, Dominyka Batkovskyte, Janey Wiggs, David S. Walton, Paula Goldenberg, Jesper Eisfeldt, Angela E. Lin, Ralph S. Lachman, Gen Nishimura, Giedre Grigelioniene

**Affiliations:** ^1^ Medical Genetics, Mass General for Children, Boston, MA, United States; ^2^Genetics Department, Cook Children´s Hospital, Fort Worth, TX, United States; ^3^Department of Molecular Medicine and Surgery, Karolinska Institutet, Stockholm, Sweden; ^4^Department of Clinical Genetics, Karolinska University Hospital, Stockholm, Sweden; ^5^Department of Pathology, Massachusetts General Hospital and Harvard Medical School, Boston, MA, United States; ^6^Department of Ophthalmology, Ocular Genomics Institute, Mass Eye and Ear Infirmary, Harvard Medical School, Boston, MA, United States; ^7^Department of Ophthalmology, Harvard Medical School, Boston, MA, United States; ^8^Department of Radiological Sciences and Pediatrics, UCLA School of Medicine, Los Angeles, CA, United States; ^9^ Department of Radiological Sciences Stanford University, Stanford, CA, United States; ^10^Orthopedic Department, International Skeletal Dysplasia Registry, UCLA School of Medicine, Los Angeles, CA, United States; ^11^ Department of Radiology, Musashino-Yowakai Hospital, Musashino, Tokyo, Japan; ^12^Endocrine Unit, Massachusetts General Hospital, Boston, MA, United States

**Keywords:** *FOXC1*, Axenfeld-Rieger Syndrome, De Hauwere Syndrome, skeletal anomalies, genome sequencing, case report

## Abstract

*FOXC1* is a ubiquitously expressed forkhead transcription factor that plays a critical role during early development. Germline pathogenic variants in *FOXC1* are associated with anterior segment dysgenesis and Axenfeld-Rieger syndrome (ARS, #602482), an autosomal dominant condition with ophthalmologic anterior segment abnormalities, high risk for glaucoma and extraocular findings including distinctive facial features, as well as dental, skeletal, audiologic, and cardiac anomalies. De Hauwere syndrome is an ultrarare condition previously associated with 6p microdeletions and characterized by anterior segment dysgenesis, joint instability, short stature, hydrocephalus, and skeletal abnormalities. Here, we report clinical findings of two unrelated adult females with *FOXC1* haploinsufficiency who have ARS and skeletal abnormalities. Final molecular diagnoses of both patients were achieved using genome sequencing. Patient 1 had a complex rearrangement involving a 4.9 kB deletion including *FOXC1* coding region (Hg19; chr6:1,609,721-1,614,709), as well as a 7 MB inversion (Hg19; chr6:1,614,710-8,676,899) and a second deletion of 7.1 kb (Hg19; chr6:8,676,900-8,684,071). Patient 2 had a heterozygous single nucleotide deletion, resulting in a frameshift and a premature stop codon in *FOXC1* (NM_001453.3): c.467del, p.(Pro156Argfs*25). Both individuals had moderate short stature, skeletal abnormalities, anterior segment dysgenesis, glaucoma, joint laxity, *pes planovalgus*, dental anomalies, hydrocephalus, distinctive facial features, and normal intelligence. Skeletal surveys revealed dolichospondyly, epiphyseal hypoplasia of femoral and humeral heads, dolichocephaly with frontal bossin gand gracile long bones. We conclude that haploinsufficiency of *FOXC1* causes ARS and a broad spectrum of symptoms with variable expressivity that at its most severe end also includes a phenotype overlapping with De Hauwere syndrome.

## Introduction

Axenfeld-Rieger syndrome (ARS) is an autosomal dominant condition characterized by a wide spectrum of anterior segment dysgenesis associated with a high risk of glaucoma and cataract development. Its extraocular findings include hypertelorism, midface and maxillary hypoplasia, hypodontia, small, cone-shaped teeth, enamel defects, heart and renal malformations, joint pain, and short stature (Reis and Semina, 2011; Chang et al., 2012; de Vos et al., 2017; Reis et al., 2022; Zhou et al., 2023). Less common features are redundant periumbilical skin and CNS abnormalities, such as cerebellar vermis hypoplasia, enlarged *cisterna magna*, and dilated ventricles (Chang et al., 2012; de Vos et al., 2017). ARS is genetically heterogeneous (MIM #180500, #601499, #602482); type 1 and 3 are caused by pathogenic variants in *PITX2* and *FOXC1* respectively, while the genetic cause of ARS type 2 is not known. Approximately 40%–70% of ARS are due to pathogenic variants in *FOXC1* (6p25.3) or in *PITX2* (4q25) (Seifi and Walter, 2018; D'Haene et al., 2011; Tümer and Bach-Holm, 2009; Reis et al., 2012). Pathogenic variants in *FOXC1* include missense, nonsense, and frameshift mutations, as well as deletions and partial duplications of the gene (Zhou et al., 2023; D'Haene et al., 2011; Tümer and Bach-Holm, 2009; Nishimura et al., 2001). Today, there are more than 400 reported patients with heterozygous disease-causing whole gene deletions, missense, or nonsense variants in *FOXC1* (Zhou et al., 2023).

Axenfeld-Rieger anomaly is part of the more severe 6p25 deletion syndrome (MIM #612582), which also includes features of intellectual disability, hypotonia, hydrocephalus, and Dandy-Walker malformation (de Vos et al., 2017). In 1973, De Hauwere *et al.* reported an autosomal dominantly inherited syndrome in a mother and her two children with Axenfeld-Rieger anomaly, iris dysplasia, hyperlaxity of the joints, hip dislocation and *coxa valga*. These patients had low muscular tone, large *sella turcica*, dilatation of cerebral ventricles and subarachnoidal cisterns, sensorineural hearing impairment, psychomotor delay, short stature, and distinctive facial features including hypertelorism, telecanthus, and maxillary hypoplasia (De Hauwere et al., 1973). Furthermore, patients with distal chromosome 6p deletions including p25.3 and *FOXC1* have been reported to have a skeletal phenotype with short stature, hip dysplasia, femoral and humoral head flattening, joint hypermobility and/or vertebral anomalies (Kannu et al., 2006; Lowry et al., 2007; Martinez-Glez et al., 2007; Reis et al., 2012). Recently, Reis et al. (2022) reported 69 individuals with ARS and pathogenic variants in *FOXC1,* 23 of whom had skeletal abnormalities. The same study also suggested that De Hauwere syndrome (DHS, 109120) is equivalent to the most severe end of *FOXC1*-related disorders.

Here, we report genetic and clinical findings in two unrelated individuals with *FOXC1* haploinsufficiency and a phenotype resembling DHS. Genome sequencing revealed that one of the patients has a complex rearrangement involving a deletion of *FOXC1,* and that the other has a single nucleotide deletion leading to a frameshift and a stop codon. Our study confirms that DHS is the most severe end of *FOXC1*-related disorders.

## Clinical findings

### Patient 1

The female patient was born at 41 weeks of gestation to a 27-year-old primigravida mother and a 41-year-old father. The pregnancy was complicated by twin gestation and first trimester twin demise. Amniocentesis for karyotype and alpha-fetoprotein level were normal. The delivery and postnatal course were unremarkable. Birth weight and length were 3.09 kg (z-score −0.31) and 50.8 cm (z-score +0.26). Initial examination revealed a hoarse cry, right scapular hemangioma, and *talipes equinovarus* which were treated with casting.

The patient was diagnosed with tracheomalacia at age 2 months. At the time, relative macrocephaly, frontal bossing, hypertelorism, depressed nasal bridge, anteverted nares, mild *pectus excavatum*, single palmar creases, and short limbs with apparent rhizomelia and upper extremity acromelia were noted. Her knees had lateral dimples bilaterally and increased anteroposterior laxity. She had mild hypotonia. Head ultrasounds at ages 4 and 5 months showed mild fullness of both lateral ventricles without frank hydrocephalus nor evidence of increased intracranial pressure. Infancy and early childhood were significant for poor growth, conductive hearing loss secondary to congenitally fused ossicles and recurrent otitis media, and diagnoses of bicuspid aortic valve and persistent superior vena cava to the coronary sinus. By age 3.5 years, height and weight had dropped significantly to 86 cm (z-score −3.2) and 11.3 kg (z-score −2.3), respectively, while head circumference remained relatively large at 51.5 cm (z-score +1.8). She had radial head subluxation, *genu valgum,* and generalized joint laxity.

She was diagnosed with visual acuity deficit and infantile glaucoma, and noted to have iris hypoplasia, an anteriorly displaced line of Schwalbe, abnormal angulation and insertion of the irises, and right-sided uveal ectropion. Eye movements and retinal examination were normal. She required multiple ocular surgeries, including bilateral surgical goniotomy, trabeculectomy, and implantation of aqueous humor drainage device to aid with intra-ocular fluid outflow. Prior to surgery, eye examination revealed 20/60 visual acuity with no refractive defects. The intraocular pressures measured 30 mm Hg bilaterally. The corneal thicknesses measured 643 μm and 678 μm. Bilateral Haab straie were present. The pupils were round and centered with a prominent inferior ectropion uvea for the right eye. On gonioscopy, the iris inserted at the level of the scleral spur. Multiple nonpigmented thin iris stromal extensions were attached to a thickened Schwalbe line circumferentially.

**FIGURE 1:**
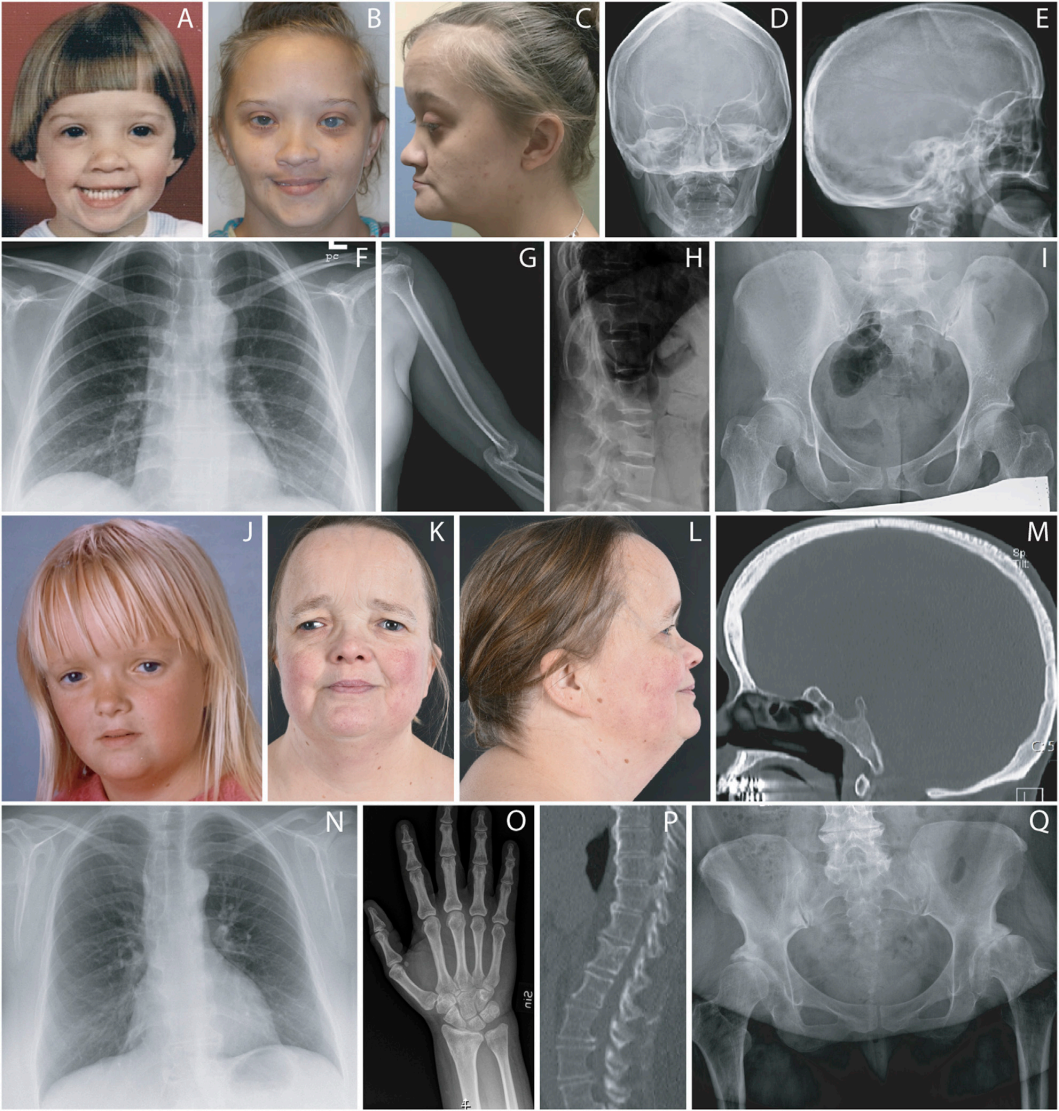
Clinical and radiographic findings at different ages **(A–C)**: Facial features of patient 1 at 4.5 and 30 years. Note broad and prominent forehead, sparse anterior hairline and medial eyebrows, prominent eyes, epicanthal folds, hypertelorism, telecanthus, depressed nasal root, bulbous nasal tip, prognathism, malar flattening, thin upper lip vermilion and mildly posteriorly rotated ears. **(D–I)**: Radiographic examination of patient 1 showed mild dolichocephaly with frontal bossing, dolichospondyly, a mildly narrow thorax, gracile long bones, and flat capital femoral epiphyses. The elbow joint was dislocated. **(J–L)**: Facial features of patient 2 at 7 and 49 years. Note hypertelorism with telecanthus, down slanting palpebral fissures, depressed nasal bridge, maxillary hypoplasia, broad and prominent forehead, mildly posteriorly rotated ears and short neck. The photographs are published with informed signed consents from both patients. **(M–Q)**: Radiographic examination of patient 2 showed dolichocephaly with frontal bossing, dolichospondyly, a narrow thorax with glenoid dysplasia, gracile ulna and radius and hip dysplasia. The computer tomography of the lumbar spine radiograms showed spondylosis and spinal canal stenosis with posterior scalloping of the vertebral bodies. The radiograms of the hip joints showed advanced degenerative joint disease along with subluxation of the left. The femoral heads were small, and the femoral necks were short.

Physical examination at age 30 showed short stature, height 143 cm (z-score −4.18) and relative macrocephaly, with a head circumference of 55.5 cm (z-score +0.12). She had generalized joint laxity, chronic joint pain and had undergone C4-C5 vertebral fusion surgery. She had ARS-typical facial characteristics (Figure 1). All her teeth were extracted due to enamel hypoplasia. There were fifth digit clinodactyly, decreased elbow mobility, mild *pectus excavatum*, calf hypotrophy, and *pes planovalgus* with long second toes. DEXA scan at age 30 years showed osteopenia in the spine (T-score −1.40) and vertebral bodies (T-score −1.80), and osteoporosis in the femoral necks (T-score −3.00).

Neurological exam revealed mild unilateral ptosis, significant vision loss (sensitive to hand motions only), and poor balance. She wears bilateral hearing aids and has undergone bilateral tympanoplasties and right ossicular prosthesis implantation. She has a high school degree, normal intelligence, and attention deficit disorder (ADD) and required special services in school due to hearing and vision impairments. She carries diagnoses of anxiety, panic disorder, and had a history of two prior miscarriages. At age 32 years, she developed seizures and started antiepileptic medication. EEG confirmed frequent epileptiform complexes over the right fronto-temporal area correlating with MRI findings of mild inferior transtentorial herniation of the medial right temporal/occipital lobe and associated mild deformity of the right cerebellum, suggestive of tentorium hypoplasia. MRI also showed mild dilation of the lateral ventricles, mild inferior displacement of the pituitary stalk and third ventricle, and several nonspecific scattered punctate foci of periventricular and subcortical T2/flair signal abnormalities.

Radiological reports at age 3.5 years, and in late childhood showed an enlarged sella turcica, dolichospondyly, overmodeling of the long bones and metacarpals, and generalized epiphyseal ossification delay as well as dysplasia of the capital femoral epiphyses. Subluxation of C4-C5, *genu valgum*, and elbow dislocation were noted. Skeletal survey at age 31 years showed mild dolichocephaly with frontal bossing, dolichospondyly with short posterior pedicles, a narrow thorax, gracile long bones, mild epiphyseal dysplasia of the proximal humeri and proximal femora and post-surgical fusion of C4-C5. Dislocation of the elbow and patellofemoral joints and *pes planovalgus* were noted (Figure 1).

### Patient 2

The patient is the first of four children born to non-consanguineous parents. Her parents are deceased, and her birth parameters and early childhood clinical records are not available. The patient’s mother suffered from glaucoma, blindness, and dental problems including dental caries and fragile teeth. She was 162 cm tall (z-score −1.26), did not have musculoskeletal complaints and died from cervical cancer at age 62. The patient has two healthy siblings, and one affected sister, who was born with anterior segment dysgenesis and atrial septal defect. The patient’s sister developed childhood glaucoma which remained undiagnosed for several years, and she is currently blind. She has dental problems, prominent forehead, and has been diagnosed with ADD and autism spectrum disorder. She is 162 cm tall (z-score −1.26) and has no musculoskeletal complaints.

The patient presented with *pubertas praecox* with menarche at age 7. She was diagnosed with hydrocephalus with pronounced enlargement of the lateral and third ventricles and a large suprasellar arachnoid cyst with bilateral compression of the middle fossa and the temporal lobes. A ventrikulo-atrial Hakim-shunt was placed when she was 9 years old. She has suffered from frequent headaches since age 30, independent of correct shunt placement or function. Her intelligence is normal, but she has difficulties recognizing faces. She has normal hearing.

Eye examination revealed alternating exotropia and congenital anterior segment dysgenesis with anterior chamber deformity, prominent strands, and atrophy of the iris stroma. She also has juvenile-onset glaucoma with excavation and paleness of the optic papilla and subsequent decrease in the visual field, discrete cataract, and arcus senilis. She has undergone bilateral glaucoma surgery with trabeculectomy at age 18 years.

At age 49, her height was 143 cm (z-score −4.18), weight 81 kg (BMI 39), and head circumference 58.5 cm (z-score +1.88). The patient has distinct craniofacial features (Figure 1) and redundant umbilical skin. She has only five remaining permanent teeth, dental implantations, and severe problems with dental caries, fragile hypoplastic teeth, and gingival bleeding. She has joint hypermobility but has never had any joint dislocations. She has *pes valgus* and pain in her feet. She underwent bilateral hip replacement at ages 39 and 40 respectively due to pain and arthrosis.

Radiographic findings of the skeleton included severe coxarthrosis, mild dolichocephaly with frontal bossing, dolichospondyly, a narrow thorax, gracile long bones, dysplastic proximal femoral epiphyses with short femoral necks, and glenoid dysplasia. The lumbar spine showed spondylosis and spinal canal stenosis with posterior scalloping of the vertebral bodies (Figure 1). Echocardiography was performed at 48 years of age with normal findings except a mild mitral and pulmonary valve regurgitation.

## Diagnostic assessment

The local Ethics Committee at Karolinska Institute approved the study (2012-2106-31/4, 2014/983-31/1), which followed the tenets of the Declaration of Helsinki.

Z-scores of the height and weight were calculated using WHO growth standards (https://www.who.int/tools/child-growth-standards/standards). For height and head circumference >5 years of age, reference tables from Fredriks et al. were used (Fredriks et al., 2000).

### Patient 1

**FIGURE 2:**
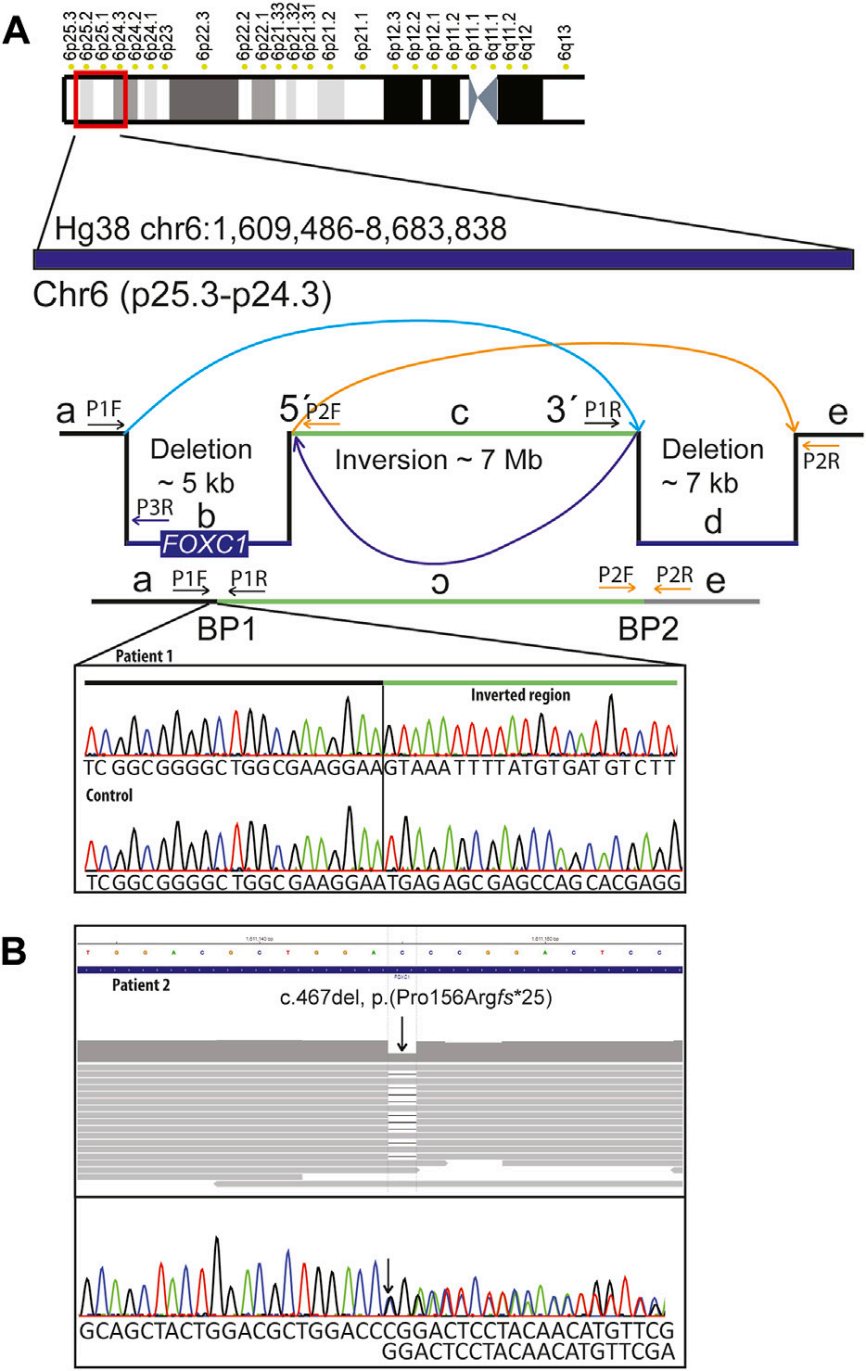
Molecular findings: **(A)** Schematic representation of the complex rearrangement and confirmation of the 5´breakpoint (BP1) using Sanger sequencing in patient 1 and in a normal control DNA sample. Complex genomic rearrangement includes: 4.9 kb deletion including *FOXC1* followed by an inversion of approximately 7 MB and a 7.1 kB deletion. Primer pairs used for amplification and sequencing of the breakpoints 1 (BP1) were P1F and P1R. For amplification of the normal control sequence corresponding to the position of BP1 we used primer pairs P1F and P3R. **(B)** Genome and Sanger sequencing of DNA sample from patient 2 showing stop-gain variant in *FOXC1* (NM_001453.3):c.467del, p.(Pro156Argfs*25).

Standard chromosome analysis at 450-band level resolution and Metaphase FISH using probes targeting the subtelomeric regions of 6p (TelVysion 6p, Abbott Molecular) and 6q (TelVysion 6q, Abbott Molecular) showed a normal female karyotype (46,XX). Array comparative genomic hybridization (aCGH) performed as described previously (Le et al., 2011) revealed an interstitial 6p25.3 deletion involving *FOXC1*. Genome sequencing (GS) (NGS; Illumina 2500, Agilent Sure Select) was performed as described previously (Lindstrand et al., 2019; Lindstrand et al., 2022) using the human genome Hg19 assembly as reference. A complex genetic rearrangement was shown with a 4.9 kb deletion (including *FOXC1* coding region) followed by a 7.0 Mb inversion and a 7.1 kb deletion (chr6:1,609,721-1,614,709del, chr6:1,614,710-8,676,899inv, chr6:8,676,900-8,684,071del). Their corresponding coordinates according to Hg38 assembly are: chr6:1609486-1614474, chr6:1614475-8676666 and chr6:8676667-8683838. The variant has been submitted to ClinVar (SUB1291463). To find the exact location of the deletion breakpoints, the region of *FOXC1* was analyzed using IGV (http://software.broadinstitute.org/software/igv/UserGuide). The breakpoint 1 was confirmed using Sanger sequencing (Figure 2A). Genome sequencing focused on 528 known skeletal dysplasia genes (Martin et al., 2019) was normal. Analysis of public HI-C datasets (http://3dgenome.fsm.northwestern.edu/) showed that the inversion disrupts multiple TADs across several tissues (Supplementary Figure S1). Further, we extracted genes within the inversion, as well as genes in the region chr6:1,000,000-11,000,00; yielding 39 genes within the inversion, 11 genes downstream, and 3 genes upstream of the inversion (Supplementary Information S1). Notably, these lists of genes include autosomal dominant disease genes, *TFAP2A* and *BMP6*. String network analysis showed a network with significantly more interactions than expected (*p* < 1.0e-16), indicating the disruption of gene clusters (Supplementary Figure S2).

### Patient 2

Array comparative genomic hybridization (aCGH) performed according to standard procedures (Pettersson et al., 2017) was normal. Genome sequencing (GS) (NGS; Illumina 2500, Agilent Sure Select) performed as described previously (Lindstrand et al., 2019; Lindstrand et al., 2022) revealed a heterozygous single nucleotide deletion in *FOXC1* Hg19 NC_000006.11(NM_001453.3): c.467del, p.(Pro156Argfs*25), (Hg38 NC_000006.12(NM_001453.3):c.467del), which was confirmed with Sanger sequencing (Figure 2B). The variant has been submitted to ClinVar (SUB12914356). The patient’s siblings were not available for segregation analysis. Skeletal dysplasia gene panel focused on 528 known skeletal dysplasia genes (Martin et al., 2019) was normal. Human Phenotype Ontology (HPO) (Köhler et al., 2014) terms Glaucoma and Axenfeld anomaly was used to find clinically relevant variants in patient 2.

## Discussion

In this report we describe two individuals with overlapping clinical features due to *FOXC1* haploinsufficiency: one with a complex rearrangement in the *FOXC1* locus and another with a nonsense variant in the same gene. In addition to ARS, both individuals showed significant skeletal abnormalities which required orthopedic surgeries.

Since the discovery of *FOXC1* in 1994 (Pierrou et al., 1994), numerous pathogenic variants have been reported in patients with ARS (Zhou et al., 2023). In some cases, protein haploinsufficiency has been attributed to distal 6p25 deletions, the genotypic and phenotypic spectrum of which have been extensively characterized elsewhere (Gould et al., 2004; Delahaye et al., 2012; Weegerink et al., 2016; de Vos et al., 2017). Previously, 6p25 deletions and skeletal abnormalities were described in 2 patients with DHS (Martinez-Glez et al., 2007; Reis et al., 2012). Literature search using terms “6p25 deletion and skeletal” identifies nine additional patients with 6p25 haploinsufficiency and variable skeletal abnormalities, including vertebral anomalies, flat femoral epiphyses, delayed bone age, dislocation of joints, and abnormal feet or hands, similar to the skeletal phenotype observed in the two individuals presented in this report (Mirza et al., 2004; Caluseriu et al., 2006; Kannu et al., 2006; Martinez-Glez et al., 2007; Martinet et al., 2008; Delahaye et al., 2012; Reis et al., 2012; Linhares et al., 2015; Walsh et al., 2017).

In 2013, Gripp et al. (2013) described a single family with a missense variant p.(Arg170Trp) in *FOXC1* and anterior segment dysgenesis in five members, three of whom also had hip dysplasia. In 2019, Siggs et al. (2019) reported that one of their 8 patients with pathogenic variants in *FOXC1* had congenital hip dysplasia. Recently, Reis et al. (2022) in a study on genotype phenotype correlation in ARS including 69 individuals with pathogenic variants in *FOXC1,* described that 23 of them had short stature, hip abnormalities, scoliosis, pectus deformity and/or joint hypermobility/pain. Five had single gene deletions, 47 had point mutations and 17 had deletions involving *FOXC1* and one or more of the neighboring genes. The authors did not find any genotype-phenotype correlation regarding how many patients with deletions versus point mutations showed skeletal abnormalities. In addition, several families showed intrafamilial variability regarding short stature and skeletal complaints, which is consistent with findings in family 2 in this report. We therefore speculate that phenotype variability may occur, at least in part, due to incomplete penetrance and variable expressivity, although an under-ascertainment of mild skeletal features cannot be excluded. Taken together, skeletal anomalies are relatively common in patients with *FOXC1* haploinsufficiency and there is a major phenotype overlap between the patients with monogenic *FOXC1* involvement and 6p25 microdeletions.

Complex structural rearrangements may cause developmental anomalies, but prior to the era of GS they often escaped detection in routine genetic investigations. It is possible that the more severe phenotype observed in patient 1 is due to the 7 MB inversion that involves several developmentally important genes. The 3D structure of the genome plays an important role in the regulation of gene expression; and there is a growing number of reports indicating its importance in rare disease genetics (Melo et al., 2020; Eisfeldt et al., 2022). Using public datasets, we found that the TAD landscape of a large number of genes may be affected by the inversion found in patient 1, which may contribute to her severe phenotype.

The putative role of *FOXC1* in bone formation was first suggested by Sasaki and Hogan (1993). They reported the cloning and expression pattern of mouse *FOXC1* homolog, *Foxc1*, in non-notochordal paraxial mesoderm (i.e., somitomeres), and in neural-crest-derived head mesenchyme (notably, in the frontonasal area anterior to the eye and in a region extending from the optic to the otic vesicles). Later, the role of *Foxc1* in the differentiation of prechondrogenic mesenchyme into cartilage was suggested by Kume et al. (1998), who showed strong *Foxc1* expression in mesenchymal cells of axial and appendicular skeleton precursors. They reported that homozygous *Foxc1* knockout mice died at birth and had anomalies of the skull, skeleton, heart, and eyes as well as hydrocephalus (Kume et al., 1998). Yoshida et al. (2015) demonstrated the role of *Foxc1* in endochondral ossification via its key interaction with Gli2 in the Indian hedgehog (Ihh)-Gli2 signaling pathway. This interaction results in the downstream expression of Ihh target genes involved in endochondral ossification, including *PTHrP* and *COL10A1*. Homozygous loss-of-function mice (*Foxc1* ^ch/ch^) showed delayed endochondral ossification and skeletal anomalies including a “slight dwarf phenotype” with short limbs, epiphyseal dysplasia and low bone mineralization (Yoshida et al., 2015). While similar functional evidence is lacking equivalent studies in human primary cells or tissues, these murine models support the biological importance of *FOXC1* in skeletal development and lend biological plausibility to the skeletal phenotype observed in DHS.

In addition to the ophthalmologic and skeletal abnormalities, other organ anomalies present in the individuals described in this study include cardiovascular anomalies, ossicular malformations, and severe dental problems with enamel hypoplasia, hypodontia, dental caries and fragile teeth. These symptoms are consistent with those of previously reported patients with *FOXC1* haploinsufficiency. The genetic diagnoses have made it evident for the patients in this study that they suffer from a rare condition, and that their symptoms that are unusual in the general population are common in this rare congenital condition. The information regarding dental anomalies in other patients with *FOXC1* haploinsufficiency was used to support the argument to acquire an additional dental care funding from the insurance company for patient 2.

Patient 1 has hearing impairment and adult-onset seizures, which are thought to be caused by white matter changes in the periventricular and subcortical regions. Only one patient with adult-onset seizures and CNS anomalies was reported previously, by Caluseriu et al. (2006). In the study by Reis et al. (2022) 15/16 individuals with *FOXC1* disruptions showed white matter abnormalities on MRI, but seizures were not reported. Similar white matter findings, ventriculomegaly, and variable Dandy-Walker anomalies have been reported in several patients with large 6p25 deletions including *FOXC1* (Lin et al., 2005; van der Knaap et al., 2006; Cellini et al., 2012; Vernon et al., 2013; Breningstall et al., 2017; de Vos et al., 2017). These patients exhibited a wide spectrum of developmental and intellectual impairments. Altogether, while the significance and pathophysiology of these lesions are poorly understood, the fact that several patients with *FOXC1* haploinsufficiency show developmental delay supports the hypothesis that FOXC1 is important for normal CNS development.

In summary, patients with haploinsufficiency of *FOXC1* may have a spectrum of anomalies ranging from isolated anterior segment dysgenesis to ARS, to a phenotype overlapping with DHS. Patients with *FOXC1*-haploinsufficiency are best served by a multisystem approach including comprehensive ophthalmologic, neurologic, otologic, audiologic, cardiac, dental, and orthopedic evaluations. Likewise, there should be a low threshold for brain imaging. We caution providers that the skeletal manifestations are likely under ascertained and may become more apparent as the patient ages. Due to the phenotype variability, definition of the follow up times is difficult, but increased awareness of the specific risks will lead to improved and personalized care. Therefore, all patients with diagnoses of apparently isolated anterior segment dysgenesis, *FOXC1*-related ARS, and 6p25 or 6p-terminal deletion syndromes should be screened for skeletal problems, and there should be a low threshold for obtaining a skeletal survey.

## Data Availability

The datasets for this article are not publicly available due to concerns regarding participant/patient anonymity. Requests to access the datasets should be directed to the corresponding author. All data analyzed at the Karolinska University laboratory cannot be shared as a whole data set according to European law (https://eur-lex.europa.eu/eli/reg/2016/679/oj). However, subsets of variants of interest can be shared upon request.
