# Supplementary material for: Case report: Extending the spectrum of clinical and molecular findings in FOXC1 haploinsufficiency syndrome
Source: Front Genet. 2023 Jun 23;14:1174046. doi: 10.3389/fgene.2023.1174046 (PMC10326848; doi:10.3389/fgene.2023.1174046)
Supplement: Supplementary file 1 [file Table1.pdf]

SUPPLEMENTARY INFORMATION 1
List of the genes with involved TADs in the rearrangement of patient 1.

TFAP2A
GCNT2
C6orf52
PAK1IP1
TMEM14C
TMEM14B
ENSG00000272162
MAK
GCM2
SYCP2L
ELOVL2
FOXQ1
FOXF2
FOXC1
GMDS
MYLK4
WRNIP1
SERPINB1
SERPINB9
SERPINB6
NQO2
RIPK1
BPHL
TUBB2A
TUBB2B
PSMG4
SLC22A23
PXDC1
FAM50B
PRPF4B
FAM217A
ECI2
CDYL
RPP40
PPP1R3G
LYRM4
FARS2
NRN1
F13A1
LY86
RREB1
SSR1
CAGE1
RIOK1
DSP
SNRNP48
BMP6

TXNDC5
BLOC1S5-TXNDC5
BLOC1S5
EEF1E1-BLOC1S5
EEF1E1
SLC35B3
