# Supplementary figures and images for: Case report: Extending the spectrum of clinical and molecular findings in FOXC1 haploinsufficiency syndrome
Source: Front Genet. 2023 Jun 23;14:1174046. doi: 10.3389/fgene.2023.1174046 (PMC10326848; doi:10.3389/fgene.2023.1174046)

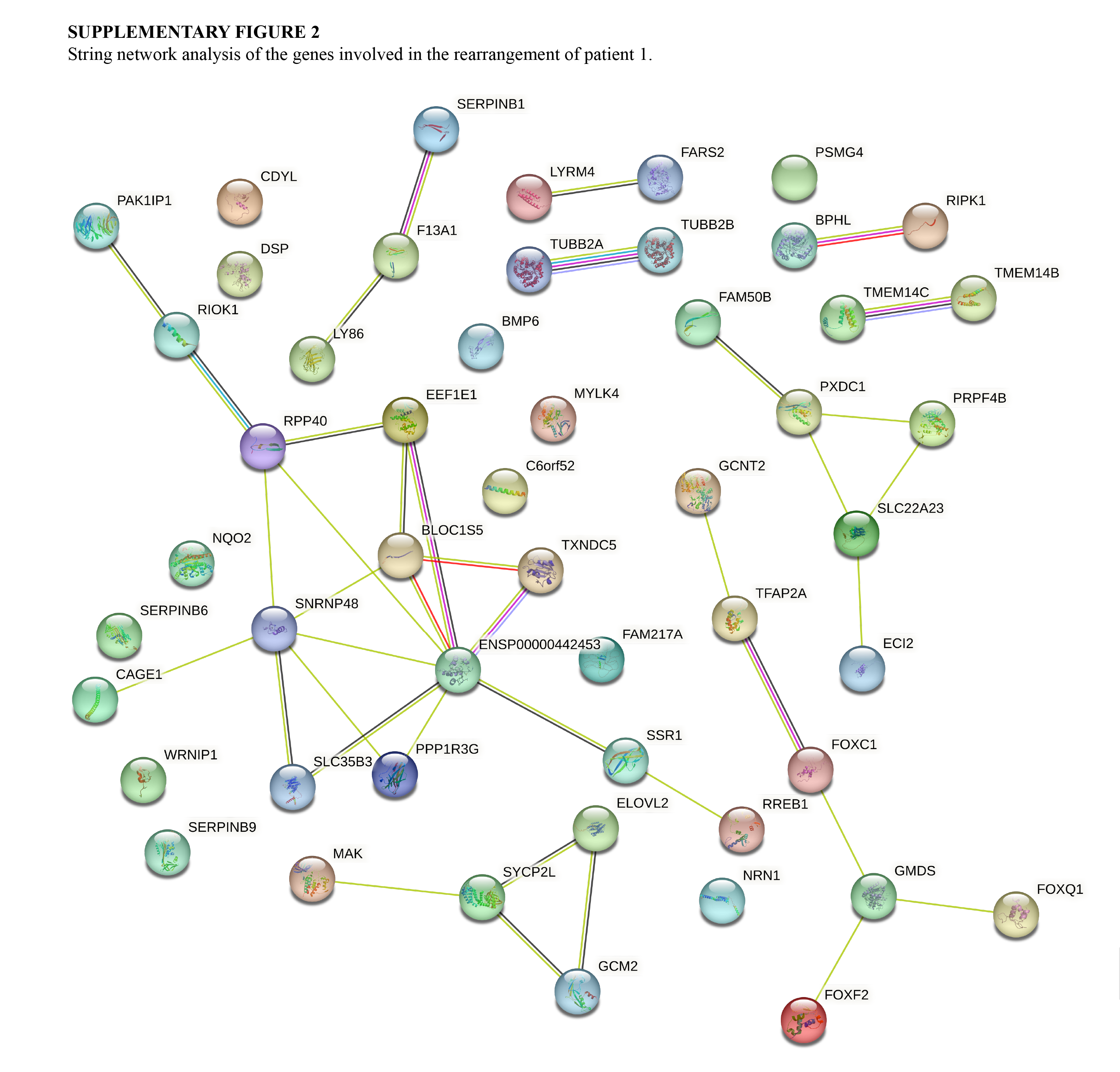

Supplement: Supplementary file 2 [file Image2.png]

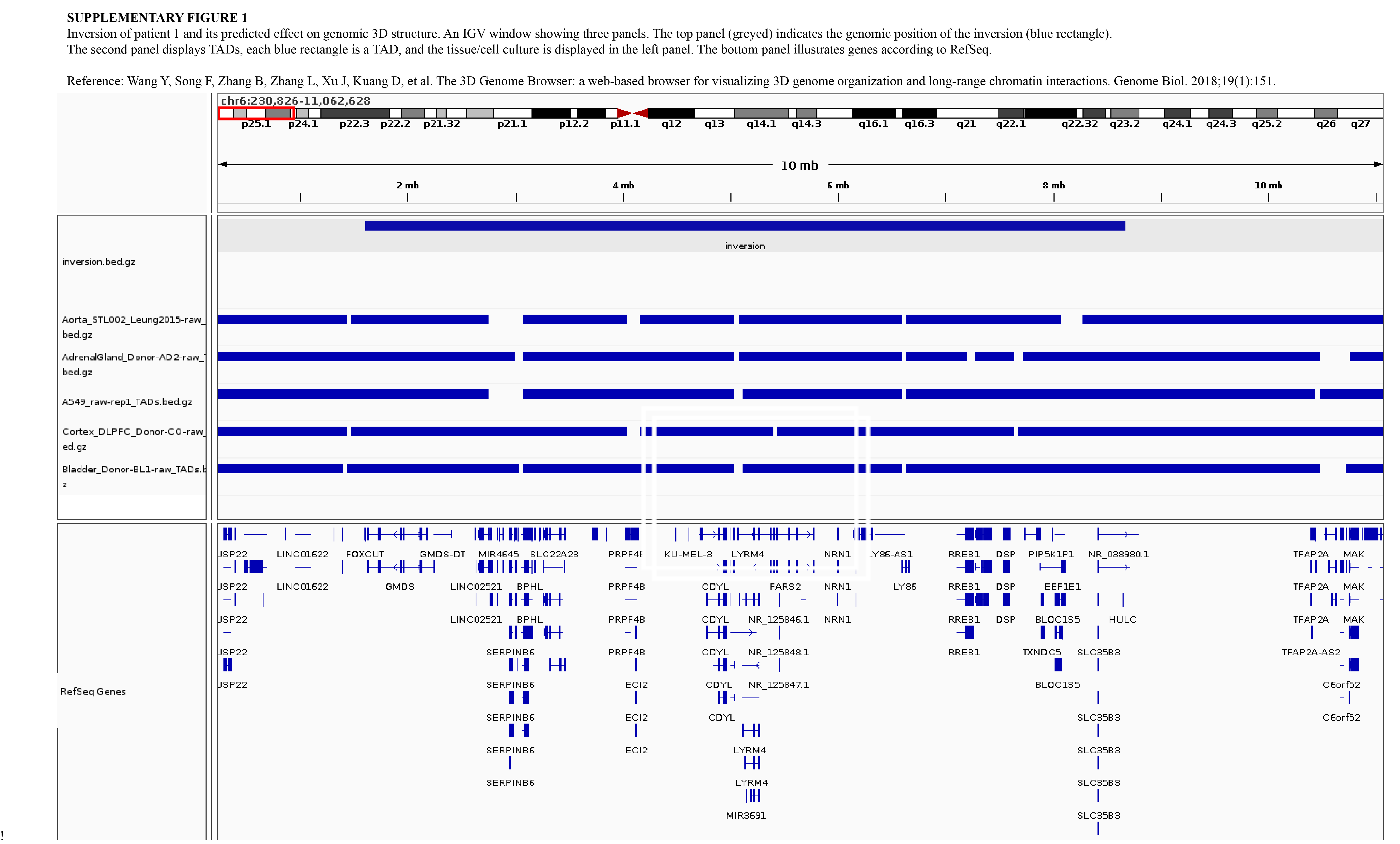

Supplement: Supplementary file 3 [file Image1.png]
